# Experimental Evaluation of Three Designs of Electrodynamic Flexural Transducers

**DOI:** 10.3390/s16091363

**Published:** 2016-08-25

**Authors:** Tobias J. R. Eriksson, Michael Laws, Lei Kang, Yichao Fan, Sivaram N. Ramadas, Steve Dixon

**Affiliations:** 1 Physics Department, University of Warwick, Coventry CV4 7AL, UK; m.laws@warwick.ac.uk (M.L.); victorkang11@126.com (L.K.); y.fan@warwick.ac.uk (Y.F.); n.ramadas@elster-instromet.com (S.N.R.); s.m.dixon@warwick.ac.uk (S.D.); 2 Elster Metering Limited, Stafford ST16 3EF, UK

**Keywords:** ultrasound, air-coupled, flexural, transducer

## Abstract

Three designs for electrodynamic flexural transducers (EDFT) for air-coupled ultrasonics are presented and compared. An all-metal housing was used for robustness, which makes the designs more suitable for industrial applications. The housing is designed such that there is a thin metal plate at the front, with a fundamental flexural vibration mode at ∼50 kHz. By using a flexural resonance mode, good coupling to the load medium was achieved without the use of matching layers. The front radiating plate is actuated electrodynamically by a spiral coil inside the transducer, which produces an induced magnetic field when an AC current is applied to it. The transducers operate without the use of piezoelectric materials, which can simplify manufacturing and prolong the lifetime of the transducers, as well as open up possibilities for high-temperature applications. The results show that different designs perform best for the generation and reception of ultrasound. All three designs produced large acoustic pressure outputs, with a recorded sound pressure level (SPL) above 120 dB at a 40 cm distance from the highest output transducer. The sensitivity of the transducers was low, however, with single shot signal-to-noise ratio (SNR)≃15 dB in transmit–receive mode, with transmitter and receiver 40 cm apart.

## 1. Introduction

Air-coupled ultrasonics is of great interest for many applications, including wireless communication [1], contactless nondestructive evaluation (NDE) [2,3], and materials characterization [4]. One of the major challenges for air-coupled transducers is the large acoustic impedance mismatch between the transducer element and air, resulting in an inefficient power transmission. Different solutions to this problem have been developed, and often make use of matching layers [5] to gradually reduce the impedance between the transducer and the propagation medium. More recently, the use of wedges with power-law taper profiles making use of the acoustic black hole effect [6] to smoothly reduce the acoustic impedance have been investigated [7,8]. The acoustic energy gets trapped—with very little reflected energy—in the tip of the wedge, which causes large amplitude vibrations.

Other available air-coupled transducers include capacitive micromachined ultrasound transducers (CMUTs) [9]) and piezoelectric micromachined ultrasound transducers (PMUTs) [10], which have excellent coupling to air and improved bandwidths. Both transduction techniques rely on cells with thin-stretched membranes, which reduces robustness and makes them unsuitable for some industrial applications.

An alternative solution is to use flexural transducers [11], which use the bending modes of a thin plate to efficiently couple to a low impedance medium, and are hence routinely used for air-coupled transduction. A common flexural transducer design that has been previously investigated [12,13] uses a thin piezoelectric disc bonded to the back of a thin, circular metal plate. As an electric field is applied across the piezoelectric element, it is distorted, causing a relatively large displacement deflection of the plate, which in turn actuates the load medium and hence generates ultrasonic compression waves.

However, flexural transducers are not without drawbacks. The bonding between the piezoelectric ceramic and the plate may degrade or even disbond over time, particularly when subjected to a hostile environment (e.g., a high temperature gas). Moreover, bonding a piezoelectric element onto a plate inevitably alters the mode shapes and frequencies of the flexing plate, and the manufacturing process of conventional flexural transducers makes it difficult to produce coherent transducers with overlapping bandwidths.

In the NDE community, electromagnetic acoustic transducers (EMATs) [14] are routinely used for non-contact transmit–receive of ultrasound waves in electrically conductive test objects. Being free of acoustic couplant and having the flexibility of transmitting and receiving multiple wave modes, EMATs are particularly attractive in some fields of non-destructive testing and non-destructive evaluation, such as detecting a sample that is in motion or at a high temperature.

In this paper, we combine the working principles of flexural transducers with EMATs. Three electromagnetically actuated flexural transducers (or Electrodynamic Flexural Transducers, EDFTs) are proposed and tested. Compared to conventional flexural transducers, EDFTs generate vibrations directly on the metal plate through electromagnetic coupling, which simplifies the manufacturing process of the transducers and offers an improved robustness for high temperature applications. Also, better consistency in the performance of the transducers can be achieved due to the elimination of the bond layer. Because of the greater flexibility in the design of the excitation coil, novel transducer geometries are made possible.

**Figure 1:**
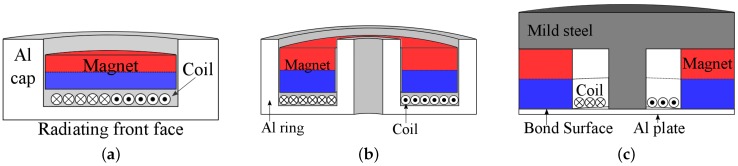
Schematic diagrams of the cross sections of the three transducer designs investigated in this paper. (**a**) Cap transducer A; (**b**) ring transducer B; and (**c**) enhanced field transducer C.

**Figure 2:**
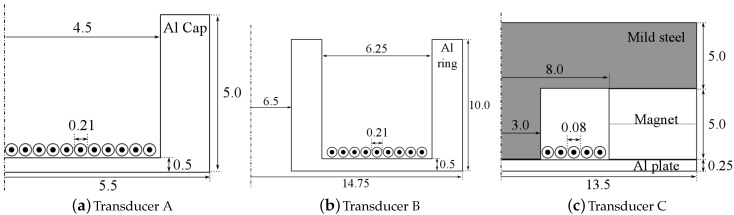
Sideview, axisymmetric, cross-sectional schematic diagrams of the three transducer designs, with all dimensions in mm.

The EDFTs investigated here are shown schematically in Figure 1. Initial results on the flexural properties of transducer A (Figure 1a) were presented in [15]. The dimensions of the three transducers are given in Figure 2.

One drawback of the EDFT is a loss of electro-mechanical efficiency compared to piezoelectrically actuated transducers, as well as the requirement of a high amplitude pulsed current, which can be an inconvenience for intrinsic safety requirements. However, for some applications, these considerations are of less importance, and the advantages makes the EDFT a relevant addition to air-coupled ultrasonic technology.

### 1.1. Electrodynamic Coupling Principles

**Figure 3:**
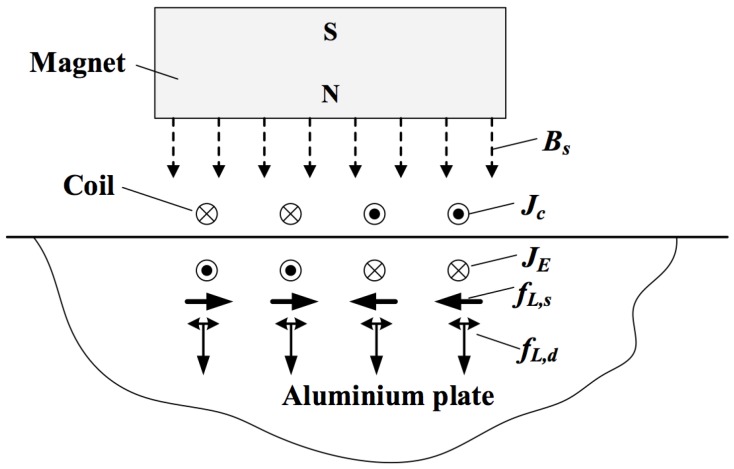
Working mechanism of an electromagnetic acoustic transducer (EMAT) on an aluminium sample. $f_{L,d}$ is the Lorentz force due to the dynamic magnetic field, and $f_{L,s}$ the Lorentz force from the static magnetic field.

The electrodynamic coupling to the front face of the EDFT is via the Lorentz mechanism in this case, as is often the dominant coupling mechanism with EMATs. Generally speaking, three mechanisms are responsible for the electro-acoustic transduction of EMATs: the Lorentz force, the magnetostrictive force, and the magnetization force [16]. An EMAT typically consists of a coil carrying an alternating current and a magnetic field generated from either permanent magnet(s), an electromagnet, or even a pulsed electromagnet. In aluminium, the Lorentz force is the only generation mechanism that needs to be considered for EMATs. The generation process of an EMAT on an aluminium plate is illustrated in Figure 3. When an alternating current $J_{c}$ travels through the coil, a dynamic magnetic field $B_{d,m}$ is generated in the electromagnetic skin depth of the aluminium plate. The time-varying magnetic field induces an eddy current $J_{E}$ within the skin depth of the plate, and the electrons that constitute this eddy current experience Lorentz forces $f_{L,d}$ and $f_{L,s}$ when the eddy current $J_{E}$ interacts with the dynamic magnetic field $B_{d,m}$ from the EMAT coil and the static magnetic field $B_{s}$ from the magnet. The directions of these forces are shown in Figure 3. The electrons that experience these Lorentz forces scatter off the atoms, exchanging momentum with the atoms. This in turn gives rise to the coherent motion of the aluminium atoms, leading to the generation of an ultrasonic wave. The resultant wave modes are determined by the configurations and the parameters of the EMAT, and the dimensions and the material properties of the plate.

In the detection process, when the atoms and free electrons in the metal move due to the displacement associated with an ultrasonic wave, the free electrons with energy above the Fermi level will experience a Lorentz force in the presence of a magnetic field. These electrons constitute a current that will generate a magnetic field, which when within the electromagnetic skin depth of the surface will produce a magnetic field external to the sample that can induce an electromotive force (voltage) on a suitably oriented detection coil [17]. An EMAT operating as a detector is a velocity sensor rather than a displacement sensor [18].

While the operational principles of the EDFT device proposed in this paper are similar to those of an EMAT, there are some subtle differences—mainly the dilation characteristics of the flexural membrane and how they affect the induced magnetic/electric fields. A complete understanding of this physical phenomenon warrants a detailed investigation, and will be reported at a later date. The main purpose of this paper is to perform an empirical study on three selected proof-of-concept EDFT device configurations.

## 2. Methods

A Polytec laser vibrometer (OFV-5000, Polytec Ltd., Harpenden, UK) was used to measure the front face displacement of the transducers, and an acoustic microphone (BK 4138-A-015, Brül & Kjær, Nærum, Denmark) to measure the acoustic pressure. The transducers were tested in receive mode by generating ultrasound from a commercial 50 kHz, air-coupled, piezoelectric transducer (AT50, Airmar Technology Corporation, Milford, CY, USA). An in-house built 20 dB EMAT amplifier was connected to the receiver transducer. Transducers B and C were also tested in transmit–receive mode 40 cm apart, with transducer B as transmitter and C as receiver.

The equations describing harmonic vibrations in thin, edge clamped, circular plates [19] were used to find the approximate mode frequencies and mode shapes of the EDTFs. Design parameters such as plate thickness and radius were chosen to give a fundamental mode resonance around 50 kHz. The frequency is appropriate for air-coupled applications over the distances considered in this paper (<1 m), as it is low enough not to be heavily attenuated. Fundamental mode vibrations are in phase across the whole radiating face, resulting in a larger net volume displacement of air for a given displacement amplitude. It is possible to operate flexural transducers in higher frequency modes, which can be preferential when a large aperture probe is required.

**Figure 4:**
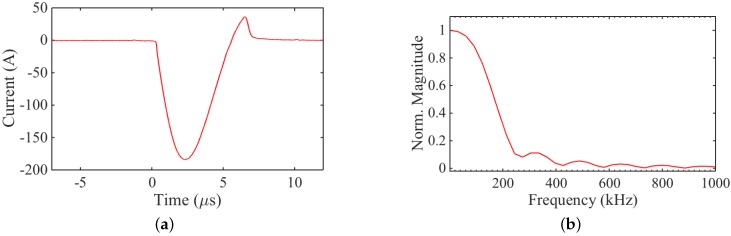
(**a**) Excitation current pulse over a small resistance (100 mΩ) in series with the transducer coil; and (**b**) frequency content of the excitation pulse.

The transducers were driven with an in-house built, wideband EMAT current pulser. The current pulse through a 100 mΩ resistor in series with the coil from transducer A is shown in Figure 4a. Although the pulser is not specifically designed to operate at 50 kHz, there is a significant amount of energy in this part of the frequency spectrum, as can be seen in Figure 4b.

## 3. Results

**Figure 5:**
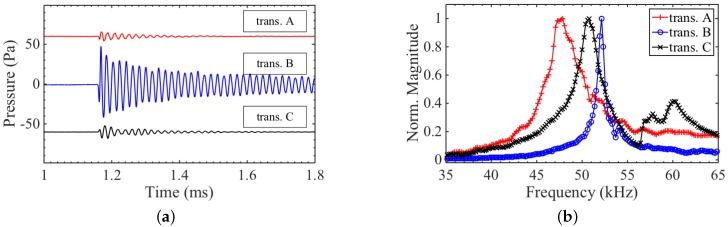
(**a**) Pressure output measured with a broadband acoustic microphone 40 cm from the transducer source. A DC offset was added to the signals from transducer A and C for better visualisation; (**b**) Frequency content of transmit signals seen in Figure 5a. The long ring down time of transducer B gives it a narrow bandwidth with a full width half maximum (FWHM) ≃1 kHz , whereas transducer A and C have a FWHM of 5 kHz and 3.5 kHz, respectively.

Generating ultrasound with an electromagnetically-excited flexural transducer is straightforward. Large pressure amplitudes were observed 40 cm from the front face of the transducers, as seen in Figure 5a. Transducer B produced the greatest pressure amplitudes around 50 Pa or SPL=128 dB. This is about an order of magnitude greater than the pressure amplitudes from the other two designs, which is largely due to the overall greater surface area of the front face of transducer B. However, it is important to remember that the aperture of transducer A and C cannot simply be increased, as this would reduce the resonance frequency. Hence, by using the ring geometry, greater flexibility in frequency and aperture size is achieved, as the inner and outer radius can be varied separately to give a specific resonance frequency.

The frequency content of the generation signals from the transducers is shown in Figure 5b. The bandwidth of transducer B is lower compared to A and C, due to the longer ring down time, on the order of a millisecond. In general, all three transducers exhibit long ring down due to the resonant behaviour and lack of mechanical or electrical damping used. In the frequency spectrum of transducer C, there is a sharp rise around 56 kHz that looks similar to a feature commented upon in [15], which was due to the presence of non-axisymmetric vibration modes.

**Figure 6:**
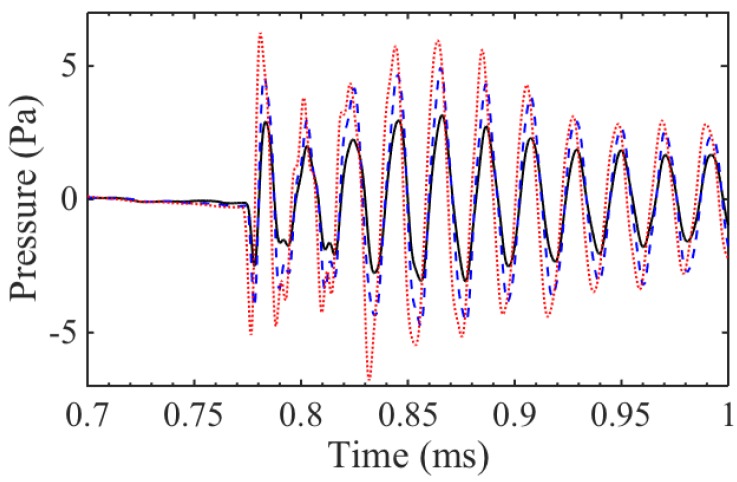
Initial pressure amplitude in air from transducer A, with no applied magnetic field (---); with an applied magnetic field setting up a Lorentz force in the same direction as the self induced repulsion force (···); and with the magnetic field in the opposite orientation, suppressing the self-induced force (—).

Figure 6 shows how the acoustic pressure produced by transducer A varies with an applied magnetic field. The direction of the static field changes the direction of the associated Lorentz force $f_{L,s}$ (see Figure 3), whereas the force $f_{L,d}$ from the dynamic field is always repulsive, regardless of the direction of the coil current $J_{c}$. The main mechanism of generation appears to be from the self-induced (dynamic) field and the associated repulsion force, as the applied magnetic field only changes the pressure amplitude by a small fraction of the amplitude for the zero field case. In contrast, for transducer C, as the direction of the current through the coil was changed, a $180^{\circ }$ phase shift was observed in the pressure wave, showing that the excitation force is a result of the Lorentz force from the static field produced by the ring magnet.

**Figure 7:**
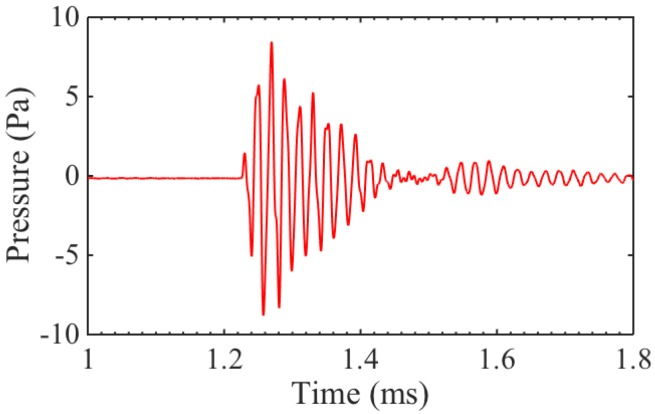
Generation pressure amplitude from commercial 50 kHz air-coupled transducer.

A static magnetic field orthogonal to the eddy current is not necessary for generating ultrasound (as seen in Figure 6), though its presence can increase the amplitude of the generated wave. However, the receiving mechanism works on a different principle, and requires a static magnetic field. The three different transducers were used to detect the signal transmitted from a commercial, large-aperture, 50 kHz transducer. The pressure amplitude of the signal at the surface of the receivers was measured with a broadband acoustic microphone to be 9 Pa, as can be seen in Figure 7.

**Figure 8:**
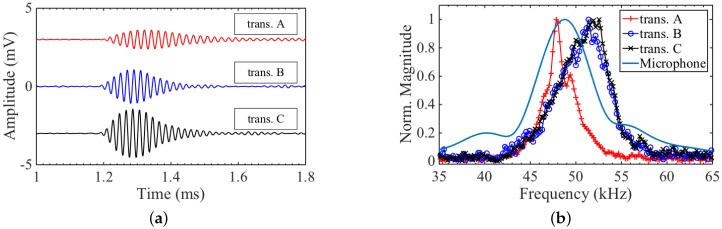
(**a**) Receive signal from the three flexural transducers. A DC offset was added to the signals from transducer A and C for better visualisation; (**b**) Frequency content from Fourier transform of receive signals shown in Figure 7 and Figure 8a. Transducers B and C have similar bandwidth, with FWHM ≃6 kHz, and transducer A has a narrower bandwidth, with FWHM ≃3 kHz.

The receive signals, with 128 averages and a low pass filter applied to reduce noise, are shown in Figure 8a. The signal amplitude is small for all three transducers, with single shot signal-to-noise ratio (SNR) ≃2 dB. It is, however, measurable, which is an important proof of concept. The largest signal comes from transducer C, which was specifically designed so that the magnetic field is oriented to enhance reception. The frequency content of the received signals of the three transducers, as well as from the microphone, are shown in Figure 8b. By comparing the received signals, it was seen that the transducers represented an accurate number of cycles, without adding much ringing. This is again seen in the frequency spectra, where the width of the 50 kHz frequency peaks from the EDFTs is comparable to that of the microphone. Since the EDFTs (and flexural transducers in general) are narrowband (high *Q*) devices, this would not be true for a broadband source. Transducers B and C do not accurately capture the centre frequency of the Airmar transducer, which is overestimated by approximately 2 kHz. This is another consequence of using a resonant system, where the transducers will skew the received signal frequency towards their mode frequency. As seen in the transmission data (Figure 8b), both transducers B and C have a mode frequency above 50 kHz, which explains the shift in centre frequency of the receive signals. Transducer A has a slightly narrower bandwidth, as it exhibited a longer ring down time, as seen in Figure 8a.

To summarize and compare the results from the three different designs, Table 1 gives for each transducer the active surface area (i.e., the area of the flexing plate), SPL, centre frequency, quality factor *Q*, and sensitivity. The sensitivity is given as received voltage per unit of acoustic pressure. The centre frequencies are taken from the transmission measurements, as these were not influenced by the characteristics of the commercial Airmar transducer.

**Figure 9:**
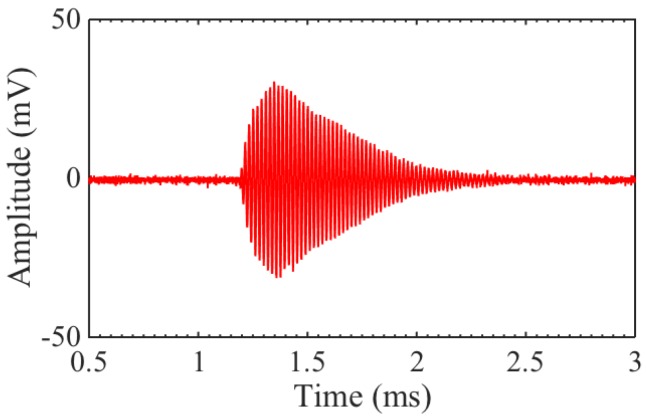
Receive signal from transducer C (through amplifier), when generating with transducer B.

As a last test, transducers B and C were set up in transmit–receive mode, with B generating and C receiving, since they produced the largest output pressure and had the best sensitivity, respectively. The distance between the transducers was roughly the same as for previous measurements, at ∼40 cm. The resulting signal with 16 averages is shown in Figure 9. The signal was clearly visible with single shot SNR ≃15 dB. The ring down time is relatively long, because both the transmitter and receiver are resonating at 50 kHz.

## 4. Conclusions

The electromagnetically-coupled flexural transducer introduces a novel design for air-coupled ultrasonic transduction. The advantage compared to standard flexural transducers is the independence from piezoelectric elements, which enables the transducer to operate at higher temperatures, reduces the number of bonds that can fail over time, and introduces new possibilities in terms of aperture design.

Of the different designs outlined and tested in this work, it has been demonstrated that the design of transducer B offered the greatest design flexibility, as it allowed a larger transducer aperture for a given operational frequency, and hence a greater pressure output. However, a long ring down resulted in a narrow bandwidth and high Q value for this transducer, which could be improved by introducing mechanical damping. Commercial piezoelectric flexural transducers often have a light backing material (e.g., cotton) behind the piezoelectric element, which dampens the oscillations without significant impact on the flexural properties of the transducer. This method should apply equally to EDFTs. Specifically for EDFTs, because of the non-contact coupling, the back of the flexing plate could be coated with an elastic material, to increase mechanical damping. Potentially, a specific quality factor could be achieved by optimizing the material and thickness of this damping layer.

The self-induced field was shown to be sufficient for the generation of ultrasonic waves, which is a significant discovery, as it allows for the construction of air-coupled electromagnetically excited ultrasound generators without the need of permanent magnets. However, it was also shown that the output pressure amplitude could easily be enhanced by the inclusion of a magnet. The dominant generation force contribution for transducers A and B is from the self-induced field. The dominant generation force for transducer C is from the static field.

The EMAT pulser used to excite the EDFTs was not optimized for flexural systems. In general, for flexural transducers, a tone-burst (multi-cycle) excitation signal is used, to make use of the resonance behaviour and high *Q* factor. The output amplitude of the EDFTs could hence be increased significantly by using an appropriately narrowband pulser.

In reception, all three transducers were able to pick up a pressure wave with 9 Pa amplitude that was produced by a large-aperture commercial air-coupled 50 kHz transducer. The SNR of the receive signal was quite low, around 2 dB for single shot data, and indicative of a low sensitivity. The results are, however, still significant as a proof of concept.

Since the reception mechanism is not the reciprocal of the generation mechanism for EDFTs, a good generator design does not necessarily make a good receiver, as was indeed seen when comparing the transmit and receive signals in Figure 5a and Figure 8a. Transducer B produced by far the largest output pressure amplitude, but recorded a slightly smaller voltage in reception for a given pressure wave compared to transducer C.

## Figures and Tables

**Table 1:** Summarized results from the three transducer designs.

Transducer	Area (${\mathrm{mm}}^{2}$)	SPL (dB)	Frequency (kHz)	Q	Sensitivity (mV/Pa)
A	63.6	109	48	10	0.07
B	436.9	128	52	26	0.12
C	172.8	112	51	10	0.18
